# Cytokinetic engineering enhances the secretory production of recombinant human lysozyme in *Komagataella phaffii*

**DOI:** 10.1186/s12934-024-02434-w

**Published:** 2024-06-18

**Authors:** Yong-Jun Zhong, Yang-Yang Luo, Haiyang Xia, Qing-Wei Zhao, Xu-Ming Mao

**Affiliations:** 1 School of Pharmaceutical Sciences, Taizhou University, Jiaojiang, Zhejiang Province 318000 China; 2 Polytechnic Institute, Zhejiang University, Hangzhou, 310015 China; 3 Institute of Pharmaceutical Biotechnology, School of Medicine, Zhejiang University, Hangzhou, 310058 China; 4 Department of Clinical Pharmacy, The First Affiliated Hospital & Institute of Pharmaceutical Biotechnology, School of Medicine, Zhejiang University, Hangzhou, 310058 China; 5 Zhejiang Provincial Key Laboratory of Plant Evolutionary Ecology and Conservation, Taizhou University, Taizhou, 318000 China; 6 Zhejiang Provincial Key Laboratory for Microbial Biochemistry and Metabolic Engineering, Hangzhou, 310058 China; 7 State Key Laboratory for Diagnosis and Treatment of Infectious Diseases, Hangzhou, 310058 China

**Keywords:** Human lysozyme, *Komagataella phaffii*, Cytokinesis, Secretory production, Genetic engineering

## Abstract

**Background:**

Human lysozyme (hLYZ) is a natural antibacterial protein with broad applications in food and pharmaceutical industries. Recombinant production of hLYZ in *Komagataella phaffii (K. phaffii)* has attracted considerable attention, but there are very limited strategies for its hyper-production in yeast.

**Results:**

Here through Atmospheric and Room Temperature Plasma (ARTP)-based mutagenesis and transcriptomic analysis, the expression of two genes *MYO1* and *IQG1* encoding the cytokinesis core proteins was identified downregulated along with higher hLYZ production. Deletion of either gene caused severe cytokinesis defects, but significantly enhanced hLYZ production. The highest hLYZ yield of 1,052,444 ± 23,667 U/mL bioactivity and 4.12 ± 0.11 g/L total protein concentration were obtained after high-density fed-batch fermentation in the *Δmyo1* mutant, representing the best production of hLYZ in yeast. Furthermore, *O*-linked mannose glycans were characterized on this recombinant hLYZ.

**Conclusions:**

Our work suggests that cytokinesis-based morphology engineering is an effective way to enhance the production of hLYZ in *K. phaffii*.

**Supplementary Information:**

The online version contains supplementary material available at 10.1186/s12934-024-02434-w.

## Background

Lysozyme is a natural antibacterial agent with broad applications in food preservation [1] and pharmaceutical industries [2]. Human lysozyme (hLYZ) is composed of 130 amino acids and contains four pairs of disulfide bonds [3]. Compared with the widely used egg white lysozyme, hLYZ has stronger protein stability and antibacterial activity, as well as lower immunogenicity [4]. Egg lysozyme can be purified from the egg white in an industrial scale. However, hLYZ is highly limited for its availability from humans, and researchers have developed several transgenic systems in animals, plants and microorganisms for the recombinant production of hLYZ [4]. Nevertheless, the production level of recombinant hLYZ remains low, resulting in higher production costs and impeding its market competitiveness and widespread uses.

*Komagataella phaffii* (*K. phaffii*), also known as* Pichia pastoris*, has been widely recognized as a robust platform for the secretory production of recombinant proteins. With its recognition as a “generally recognized as safe” (GRAS) organism, this yeast has been extensively utilized in pharmaceutical and food industries [5, 6]. To improve the production of recombinant hLYZ in *K. phaffii*, several strategies have been employed, including optimization of signal peptides [7] and gene dosage [8], enhancing the capacity of protein folding and processing through co-expression of chaperones or transcription factors [8–10], optimization of methanol metabolism by developing slow methanol-utilizing strains [9], and optimization of the fermentation process [11, 12]. Notably, combination of above strategies has synergistically led to additionally improved hLYZ activity and total protein concentration to 352,000 ± 16,696.5 U/mL and 3.18 g/L, respectively [10]. Nevertheless, the limited genetic engineering strategies in *K. phaffii* have impeded further improvements of hLYZ production.

Morphology engineering has been demonstrated as a new promising strategy to develop more efficient microbial cell factories to improve bioproduction yield [13, 14]. This strategy aims to modify cell shape and division patterns by manipulating genes related to cell morphology [14]. Cytokinesis is essential for the survival of all cellular organisms as it mediates the separation of mother and daughter cells during cell cycle [15]. In budding yeast, cytokinesis is driven by two interdependent cellular events: the constriction of actomyosin ring and the formation of primary septum [16]. Manipulating cytokinesis-related genes in bacteria has achieved bacterial morphology engineering [14]. For example, the bacterial gene *ftsZ*, which encodes the key component of the Z-ring, directly affects Z-ring assembly and cytokinesis through its overexpression or inhibition, thereby modifying cell morphology [14]. Similar to the function of the Z-ring in bacteria, the actomyosin ring in budding yeast plays a crucial role in driving membrane ingression at the mother-bud neck during cytokinesis, ultimately leading to efficient cell division [16, 17]. Disruption of the actomyosin ring through deletion of the core cytokinesis genes *MYO1* and *IQG1* cause notable changes in budding yeast cell morphology [18, 19], and may even result in cell lethality in some budding yeast strains [16, 20]. To date, reports on morphology engineering in *K. phaffii* are scarce, especially regarding its application in the efficient production of recombinant proteins.

Here a hLYZ-producing *K. phaffii* strain was developed with Ost1-pro signal peptide, multiple gene copies and *PDI1* co-expression. Two cytokinesis-related genes *MYO1* and *IQG1* were screened out to be involved in hLYZ production after ARTP mutagenesis and transcriptomic assays. Loss of these genes led to the cytokinesis defect, while the highest hLYZ enzymatic activity of 1,052,444 ± 23,667 U/mL and total protein concentration of 4.12 ± 0.11 g/L were obtained even after single deletion of *MYO1*. Furthermore, the glycosylation of the recombinant hLYZ in yeast was characterized. To our knowledge, this represents the highest yield of recombinant hLYZ in yeast, making our new strategy promising for hyper-production of hLYZ after further exploration and optimization of yeast cytokinesis.

## Results

### Identification of target genes based on combinatory ARTP mutagenesis and transcriptomic analysis

The initial *K. phaffii* strain LYZ-C1 producing hLYZ was developed, where the hLYZ gene was codon optimized for yeast and expressed with an optimized signal peptide Ost1-pro [21] for secretion, as well as multiple copy integration of the expression cassette. Moreover, *PDI1* gene was co-expressed to promote the proper formation of disulfide bonds within hLYZ. Based on the above routine genetic engineering, this initial LYZ-C1 strain can produce secretory hLYZ at a level of 30,888 ± 1137 U/mL after fermentation in shaking flasks for 96 h.

**Fig. 1:**
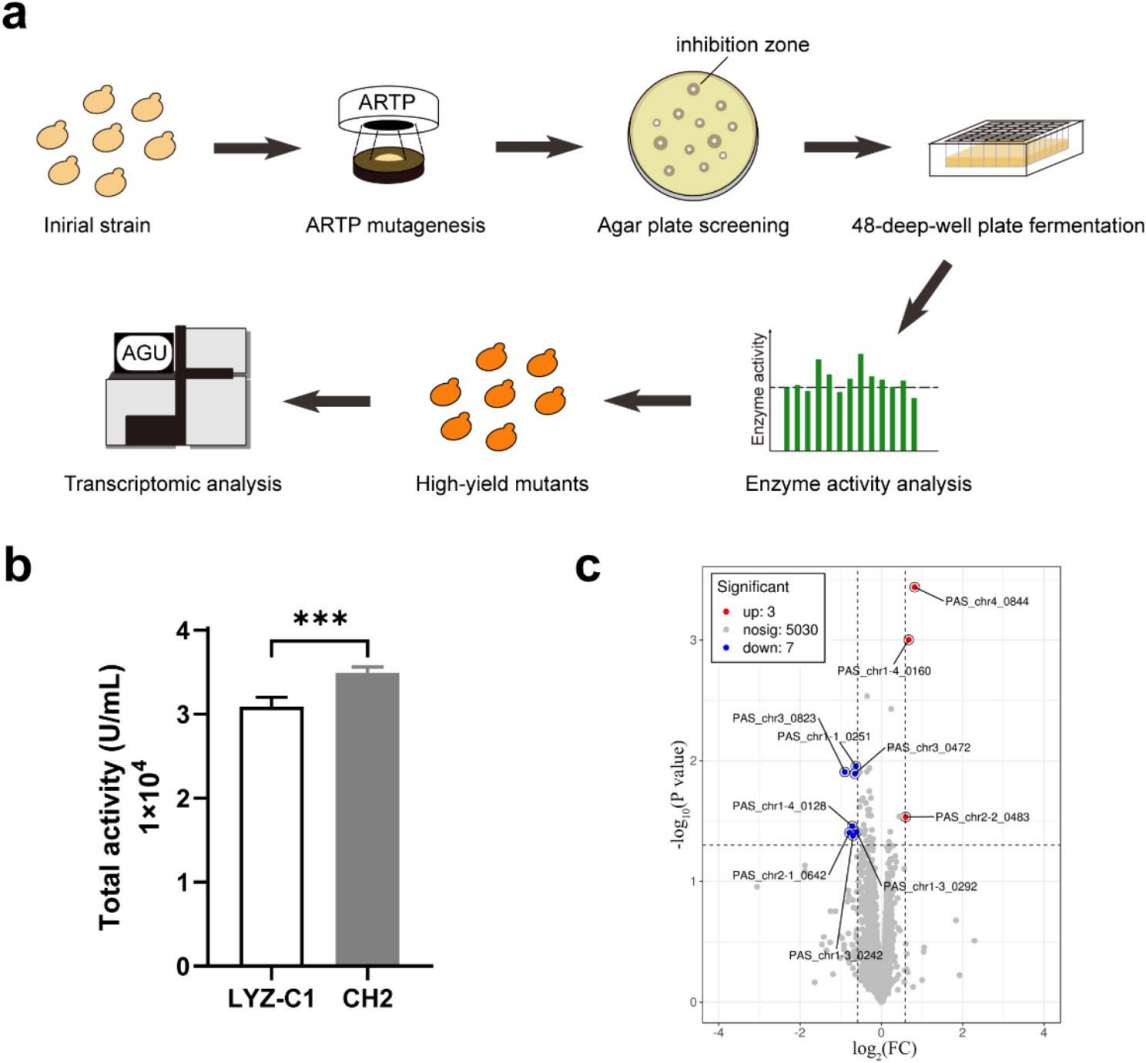
ARTP mutagenesis screening and transcriptomic analysis. (**a**) Schematic of ARTP mutagenesis, high-yield mutant screening, and analysis workflow. ARTP mutagenesis was applied to the initial strain to generate a mutant library. These mutants underwent a high-throughput bioactivity-guided screening against *Micrococcus lysodeikticus* on agar plates, followed by a secondary screening in the 48 deep-well plates and hLYZ activity assay. Transcriptomic analysis was conducted to identify potential genes related to the high-yield phenotype. (**b**) hLYZ bioactivity of the ARTP mutant strain CH2 and the initial strain LYZ-C1. The strain LYZ-C1 served as the control. Data are presented as the means ± standard deviation. ***p < 0.001. (**c**) Volcano plot of differential expressed genes in the strain CH2 compared to the reference strain LYZ-C1. Red dots, significantly upregulated genes. blue dots, significantly downregulated genes. Light grey dots, genes with no significant differences

In order to identify potential genes for further target engineering for the hyper-production of hLYZ, the strain LYZ-C1 was subjected to Atmospheric and Room Temperature Plasma (ARTP) mutagenesis (Fig. 1a), which has been widely used for efficient mutagenesis during strain development. Subsequently, a high-throughput bioactivity-guided screening against *Micrococcus lysodeikticus* was performed on the agar plates, followed by a secondary screening in the 48 deep-well plates and hLYZ activity assays (Fig. 1a). As a result, a mutant strain CH2, exhibiting a 13.1% increase of hLYZ bioactivity, was successfully obtained (Fig. 1b).

Efficient secretory production of hLYZ is a complex regulatory process. Nevertheless, the improved hLYZ production should at least result from decreased/increased expression of some negative/positive regulatory networks. Based on this assumption, transcriptomic analysis was conducted on the mutant strain CH2 and the reference strain LYZ-C1, in attempt to identify genes related to the regulation of hLYZ production. Genes showing changes in transcript levels greater than 1.5-fold and a P-value less than 0.05 were selected for downstream investigation. A total of 10 differentially expressed genes were identified (Fig. 1c). Interestingly, two cytokinesis core genes *MYO1* (PAS_chr3_0823) and *IQG1* (PAS_chr1-4_0128) were identified to be downregulated at the transcriptional levels (Fig. 1c), suggesting that yeast cytokinesis might play vital roles on hLYZ production, which has not been reported yet. Given that cell morphology engineering is a novel and promising strategy for bio-production [13, 14], understanding of the roles of cytokinesis on hLYZ production would expand the research for mechanistic investigation and genetic engineering for the hyper-production of hLYZ, as well as potentially for other eukaryotic proteins.

### Enhancing hLYZ production through cytokinetic engineering

**Fig. 2:**
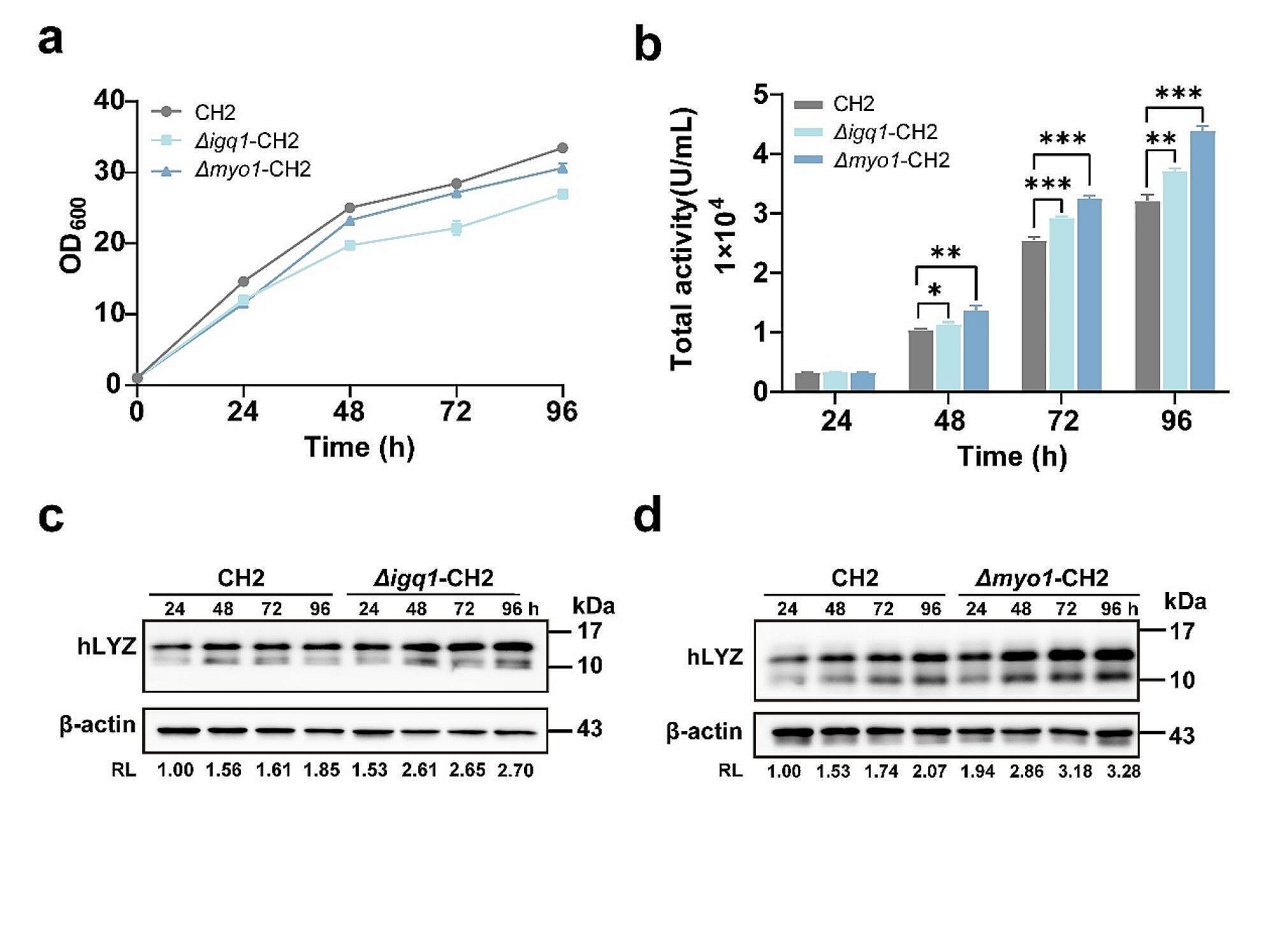
The effect of cytokinetic engineering on hLYZ production. The growth curves (**a**) and hLYZ bioactivity (**b**) of strains CH2, *Δiqg1*-CH2 and *Δmyo1*-CH2 by shaking flask fermentation. (**c**, **d**) Western blotting detection of intracellular hLYZ. Total cell lysates prepared from cells as depicted in Fig. 2b were subjected to Western blotting using an anti-hLYZ antibody, with β-actin used as the loading control. RL, the relative level of intracellular hLYZ. *p < 0.05, **p < 0.01, *** *p* < 0.001

The *MYO1* gene encodes the myosin-II protein, a critical constituent of the actomyosin ring [17]. Deletion of the *MYO1* gene disrupts the actomyosin ring, leading to significant cytokinesis defects but not cell lethality in most *S. cerevisiae* strains with various genetic backgrounds [19]. The *IQG1* gene plays a crucial role in actomyosin ring constriction and primary septum formation during budding yeast cytokinesis, and is indispensable for cell viability, as its deletion leads to cell lethality in most *S. cerevisiae* strains [16, 20]. To explore their roles for hLYZ production in *K. phaffii*, both genes were deleted individually in strain CH2. Surprisingly, unlike the observations in *S. cerevisiae*, deletion of *MYO1* or *IQG1* in *K. phaffii* did not lead to cell lethality. As shown in Fig. 2a, although deletion of *MYO1* or *IQG1* led to a slight decrease in cell growth (8.5% and 19.5% reduction at 96 h, respectively), both mutants consistently exhibited significantly enhanced hLYZ bioactivity in the supernatant. Specifically, *Δiqg1*-CH2 strain demonstrated an increase of 9.3%, 14.7%, and 15.6% in hLYZ bioactivity at 48, 72, and 96 h (Fig. 2b), respectively; while the *Δmyo1*-CH2 strain showed an increase of 32.3%, 27.5%, and 36.8% (Fig. 2b), respectively. However, the double mutant *Δmyo1Δiqg1-*CH2 did not show additional increase in hLYZ bioactivity compared to single mutant *Δmyo1*-CH2 (Figure S1). These data suggested that the *Δmyo1*-CH2 strain is a more favorable engineered strain. Given that the CH2 strain is an ARTP-mutant strain, the *IQG1* and *MYO1* genes were individually deleted in the wild-type strain LYZ-C1. As shown in Figure S2, the deletion of *IQG1* or *MYO1* in LYZ-C1 also enhances the production of hLYZ, consistent with the results seen in CH2. Moreover, three cytokinesis-regulated genes *HOF1*, *INN1* and *CYK3*, whose expression was not significantly altered in our transcriptomic assay, were also individually deleted in CH2. However, no significant improvement of hLYZ bioactivity was observed in the *Δhof1*, *Δinn1* or *Δcyk3* mutant (Figure S3). These findings suggest that *K. phaffii MYO1* and *IQG1* are promising targets for genetic engineering to enhance protein production.

The increased total extracellular bioactivity of hLYZ in both mutants might result from promoted biosynthesis and/or enhanced secretion of this lysozyme. Next, the production of intracellular hLYZ was examined to investigate this possibility. Total cell lysates were prepared from above three strains at different time points (as shown in Fig. 2c and d) and subjected to Western blotting with an anti-hLYZ antibody. Our results showed that the molecular weight of the major intracellular hLYZ was 15 kDa in all time points (Fig. 2c and d). Importantly, the relative levels of the intracellular 15 kDa hLYZ were higher in *Δiqg1*-CH2 and *Δmyo1*-CH2 compared to CH2 (Fig. 2c and d). In *Δiqg1*-CH2, the increased level of intracellular 15 kDa hLYZ ranged from 46 to 67%, while in *Δmyo1*-CH2, it ranged from 58 to 94% compared to CH2. Thus, our data suggested that deletion of *MYO1* or *IQG1* is beneficial for the biosynthesis of hLYZ in *K. phaffii*, consequently improving the apparent production yield of this lysozyme.

### Production of hLYZ in high-density fermentation and characterization of lysozyme glycosylation

**Fig. 3:**
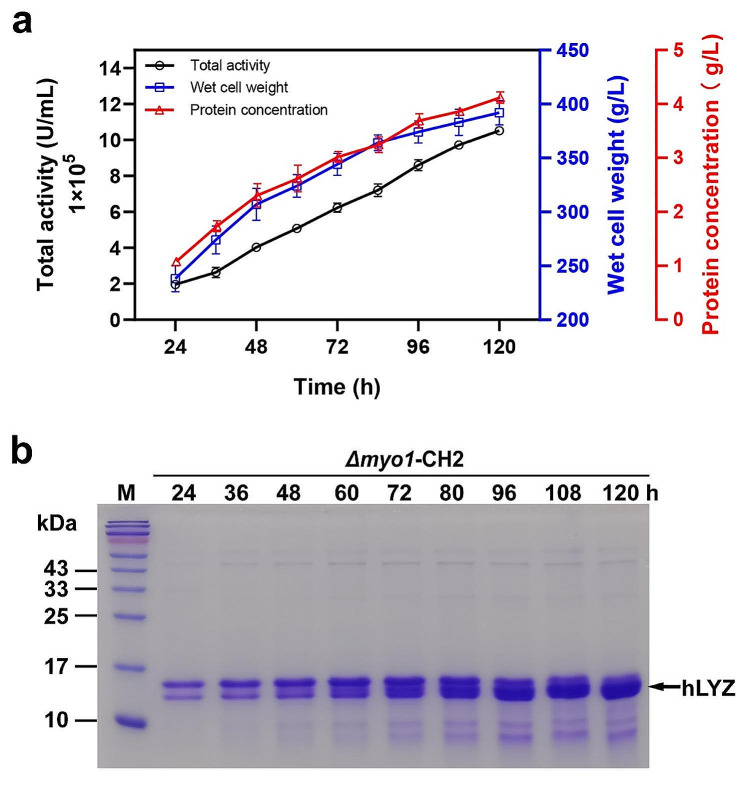
Characterization of *Δmyo1*-CH2 in high-density fermentation. (**a**) Fed-batch fermentation. The fermentation of the strain *Δmyo1*-CH2 was conducted in a 5 L bioreactor using BSM medium. The hLZY bioactivity, total protein concentration in the supernatant, and wet cell weight were assessed. (**b**) SDS-PAGE analysis of the fermentation supernatant. An equal volume of 1.6 µL of the supernatant was sampled, separated using a 15% SDS-PAGE gel, and then stained with Coomassie brilliant blue

Next, high-density fermentation was performed to further investigate the production potential of hLYZ in strain *Δmyo1*-CH2, which has the best production capacity in our shaking flasks. Throughout the fermentation process in a 5 L bioreactor with BSM medium, both the hLYZ activity and the total protein concentration increased steadily along with cell growth (Fig. 3a). After 120 h of fermentation, the wet cell weight reached 392 ± 11 g/L, and the hLYZ activity and total protein concentration in the supernatant reached 1,052,444 ± 23,667 U/mL and 4.12 ± 0.11 g/L, respectively (Fig. 3a). This represents an approximately 2.9-fold increase compared to the previously reported highest enzymatic activity of 352,000 ± 16,696.5 U/mL in a 5 L high-density fermentation [10]. Additionally, SDS-PAGE results showed a continuous accumulation of the hLYZ protein as the fermentation progressed (Fig. 3b), which was consistent with the enzymatic activity results in Fig. 3a. Overall, these results demonstrate the robustness of the engineered strain *Δmyo1*-CH2 for hLYZ production in high-density fermentation.

Interestingly, Coomassie brilliant blue staining of the fermentation supernatant proteins after SDS-PAGE revealed two closely adjacent protein bands. The lower band had a molecular weight of approximately 15 kDa (Fig. 3b), which matches the theoretical molecular weight of hLYZ, while the upper band had a molecular weight of around 16 kDa (Fig. 3b), exceeding the theoretical molecular weight of hLYZ. Western blotting data showed that both bands could be recognized by the hLYZ-specific antibody (Figure S4), suggesting that both bands represent hLYZ and that the 16 kDa band may result from protein post-translational modification. Considering the potential glycosylation modifications of proteins expressed in *K. phaffii*, it is hypothesized that the 16 kDa band may result from *N*- and/or *O*-linked glycosylation, thus leading to an increase in molecular weight. To investigate the modifications of the 16 kDa hLYZ, this protein sample was enzymatically digested with PNGase-F to remove *N*-linked glycosylation. However, this treatment did not convert the 16 kDa band to the 15 kDa band (Figure S5), indicating that *N*-linked glycosylation might not occur on hLYZ, which was consistent with the prediction of *N*-linked glycosylation for hLYZ (Figure S6). Meanwhile, the 16 kDa band was excised and subject to LC-MS/MS analysis. The analysis successfully detected *O*-linked glycans composed of different numbers of mannose residues (up to 6) at positions Ser51 and Thr52 (Figure S7), confirming that the observed 16 kDa hLYZ resulted from *O*-linked glycosylation.

### Deletion of *MYO1* and *IQG1* exhibit cytokinesis defect

**Fig. 4:**
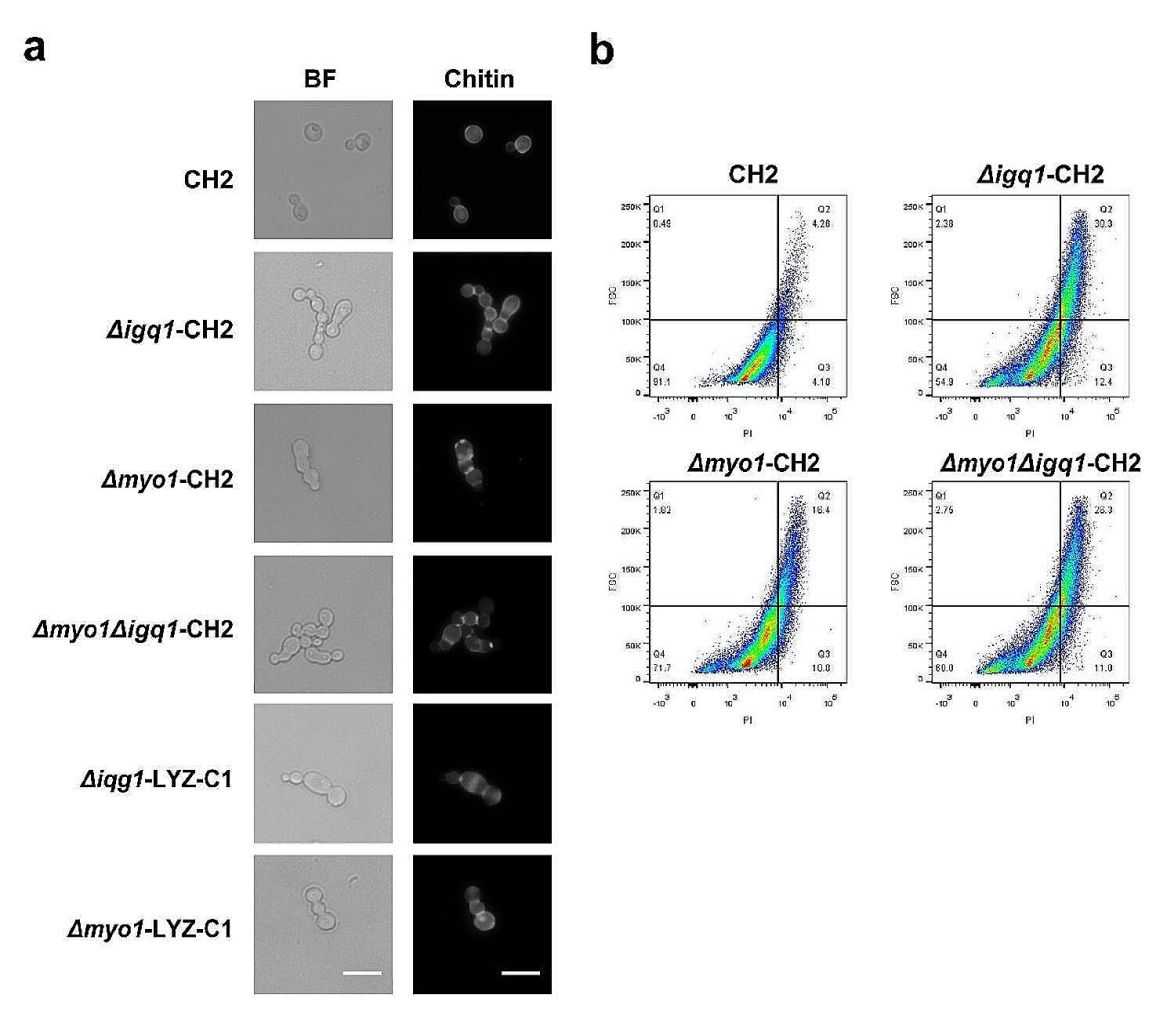
Morphological changes upon deletion of *MYO1* and *IQG1*. (**a**) Microscopic examination of yeast cell morphology. Chitin was stained with Calcofluor White. BF, Bright field. Scale bar, 10 μm. (**b**) Flow cytometry analysis. For each quadrant, the relative distribution of cells is indicated in % from total, with quadrant Q4 representing normal shaped cells with one or no bud, and quadrant Q2 representing morphology changed cells with more than one bud. Cells (> 10^5^) were counted in each case

The Myo1 protein of *K. phaffii* is composed of 1859 amino acids and shares approximately 33.12% sequence identity to the Myo1 protein of *S. cerevisiae*. Similarly, the Iqg1 protein of *K. phaffii* consists of 1578 amino acids and exhibits approximately 23.72% sequence identity to the Iqg1 protein of *S. cerevisiae*. Interestingly, unlike its counterpart in *S. cerevisiae*, Iqg1 protein in *K. phaffii* is found to be nonessential (Fig. 4a). To investigate the cytokinetic phenotypes of strains that lacking *MYO1* or *IQG1*, yeast cells were stained with Calcofluor White for microscopic chitin examination. As shown in Fig. 4a, deletion of *MYO1* and/or *IQG1* in *K. phaffii* resulted in abnormal cell morphology. These cells continue to divide and form buds but fail to separate completely, indicating cytokinesis defects similar to those observed in *S. cerevisiae* [22]. These findings suggest that Myo1 and Iqg1 play an important role in efficient cytokinesis in *K. phaffii*, similarly to their counterparts in *S. cerevisiae*. Furthermore, FACS analysis revealed a notable increase in the percentage of cells with abnormal morphology upon the deletion of *MYO1* and/or *IQG1* (Fig. 4b). Despite the severe cytokinesis defects, the individual deletion of *MYO1* and/or *IQG1* still allowed some cells to complete cytokinesis, indicating the existence of an actomyosin ring-independent cytokinetic mechanism in *K. phaffii*. Interestingly, this actomyosin ring-independent cytokinesis has also been reported in *S. cerevisiae* [23] and *C. albicans* [24]. Additionally, simultaneous deletion of *MYO1* and *IQG1* did not result in an additional increase in the percentage of abnormal cells (Fig. 4b), which suggested that Iqg1 is not necessary for actomyosin ring-independent cytokinesis in *K. phaffii*, consistent with observations in *C. albicans* [24]. However, these phenotypes were in contrast to those in *S. cerevisiae*, where Iqg1 is proposed to play a crucial role on actomyosin ring-independent cytokinesis [23].

## Discussion

The protein hLYZ holds significant promise for antimicrobial applications in the food and pharmaceutical industries; however, the current methods for increasing its recombinant expression are limited. Following the generation of the initial strain, we achieved efficient hLYZ expression through ARTP mutagenesis and targeted cytokinetic genetic engineering. The bioactivity in the supernatant of fed-batch fermentation reached 1,052,444 ± 23,667 U/mL, marking the highest ever reported yield to date and establishing the foundation for further iterative enhancement of hLYZ production in *K. phaffii*.

The potential impact of *O*-glycosylation on yeast-produced recombinant proteins is gaining increased attention [25]. Given the significant differences in glycan structures and chain length between yeast-type *O*-glycosylation and human-type *O*-glycosylation, researchers are particularly interested in whether yeast-type O-glycosylation affects the immunogenicity of recombinant therapeutic proteins. A previous study has shown that a recombinant protein with a single mannose modification produced by *K. phaffii* does not induce an immunogenic reaction [26]. Additionally, another study provided experimental evidence that *O*-glycosylation does not have a significant effect on the immunogenicity of recombinant human platelet-derived growth factor-BB (PDGF-BB) produced in *K. phaffii* [27]. These results might be attributed to the shorter *O*-glycosylation chains (typically containing no more than 6 mannoses per glycan chain) present on the recombinant proteins produced by *K. phaffii* [28]. Moreover, *O*-glycosylation has been reported to enhance the protein’s hydrophilicity [29], thereby reducing its tendency to aggregate at high concentrations. Therefore, further research is needed to investigate the effects of *O*-glycosylation on the immunogenicity of recombinant hLYZ produced by *K. phaffii*, as well as the *O*-glycosylation modification process and the protein-*O*-mannosyltransferase genes involved during the expression of the recombinant hLYZ in *K. phaffii*.

Morphology engineering is a promising strategy to improve protein production [30]. In bacteria, morphology engineering provides larger cell volumes, leading to increased accumulation of bioproducts [14]. Meanwhile, in filamentous fungi, morphology engineering amplifies the productivity of various enzymes in submerged cultures [30]. Cytokinesis studies in the budding yeast *S. cerevisiae* have established a model to investigate this fundamental process, owing to the conservation of core components and mechanisms between yeast and animal cells [15]. Prior research in budding yeast has specifically concentrated on understanding the mechanics and regulation of pivotal cytokinesis events, such as the assembly, constriction, and disassembly of the actomyosin ring, septum formation, and their spatiotemporal coordination [15]. Nevertheless, the effect of cytokinesis on the expression and secretion of recombinant proteins in budding yeast has not been clearly reported. In this study, we proposed to employ cytokinesis for morphology engineering in *K. phaffii*, thereby achieving hyper-production of recombinant hLYZ. Future research may explore morphology engineering from other perspectives to further enhance the expression levels of recombinant hLYZ. Furthermore, elucidating the molecular mechanisms by which cytokinesis-based morphology engineering enhances protein production could open new research avenues and provide theoretical foundations for optimizing *K. phaffii*-based microbial cell factories to improve efficiency.

## Conclusions

The hyper-production of hLYZ in *K. phaffii* was achieved by a combination of ARTP mutagenesis, transcriptomics, and cytokinetic engineering. Utilizing high-density fermentation using BSM medium in a 5 L bioreactor, the hLYZ bioactivity reached 1,052,444 ± 23,667 U/mL, with a total protein concentration of 4.12 ± 0.11 g/L. This represents the best production yield of hLYZ in yeast. Furthermore, *O*-linked mannose glycans in this recombinant hLYZ were characterized. These results indicate that cytokinesis-based morphology engineering holds great potential for iterative improvements and engineering of *K. phaffii* based microbial cell factories to enhance the yield of recombinant proteins.

## Methods

### Strains and culture media

*Escherichia coli* was cultivated in LB medium (0.5% yeast extract, 1% tryptone, and 1% NaCl). *K. phaffii* strains utilized in this study were detailed in Table S1. The *K. phaffii* strains were cultured for regular growth in YPD medium (1% yeast extract, 2% tryptone, and 2% glucose). MD plates (2% glucose, 1.34% yeast nitrogen base, 4 × 10^− 5^% biotin, 2% agar) or MGY medium (1% glycerol, 1.34% yeast nitrogen base, 4 × 10^− 5^% biotin) containing suitable nutrients were employed for auxotroph selection. YPM plates (1% methanol, 2% tryptone, 1% yeast extract, and 1.5% agar) were utilized for agar plate antimicrobial screening. BMGY (1% glycerol, 1% yeast extract, 2% tryptone, 1.34% yeast nitrogen base, 4 × 10^− 5^% biotin, 100 mM potassium phosphate, pH 6.0) served as the medium for seed culture during fermentation in the shaking flask, while the production of hLYZ was conducted using the BMMY medium (0.5% methanol, 1% yeast extract, 2% tryptone, 1.34% yeast nitrogen base, 4 × 10^− 5^% biotin, 100 mM potassium phosphate, pH 6.0). During fed-batch fermentation, YPG medium (consisting of 2% glycerol, 2% tryptone, 1% yeast extract) was employed for seed culture in the shaking flask, and BSM medium (0.93 g/L CaSO_4_, 18.2 g/L K_2_SO_4_, 14.9 g/L MgSO_4_·7H_2_O, 4.13 g/L KOH, 26.7 mL/L H_3_PO_4_, 40.0 g/L glycerol) supplemented with 4.35 mL/L PTM1 trace salts (6 g/L CuSO_4_·5H_2_O, 0.08 g/L NaI, 3 g/L MnSO_4_·H_2_O, 0.2 g/L Na_2_MoO_4_·2H_2_O, 0.02 g/L H_3_BO_3_, 0.5 g/L CoCl_2_, 20 g/L ZnCl_2_, 65 g/L FeSO_4_·7H_2_O, 0.2 g/L biotin, and 5 mL/L H_2_SO_4_) was utilized for the fed-batch fermentation process.

### Plasmid construction

The plasmids and primers used in this study have been documented in the Supplementary Information (Table S1 and Table S2). The Ost1-pro-hLYZ sequence was codon-optimized and synthesized by Tsingke (Hangzhou, China), and can be accessed for download from the website at https://ngdc.cncb.ac.cn/genbase/, using the accession number C_AA053474.1. Primers hLYZ-F/hLYZ-R were used to amplify Ost1-pro-hLYZ fragment from synthetic DNA, and assembled into *EcoR*I/*Kpn*I digested pPink-HC to give rise to plasmid pPinkHC-hLYZ. Primers PDI1-F/PDI1-R were used to amplify PDI1 fragment from GS115 genomic DNA, and assembled into *BamH*I/*Not*I digested pPIC9k to give rise to plasmid pPIC9k-PDI1. Primers sgRNA-MYO1-f/sgRNA-MYO1-r, sgRNA-IQG1-f/sgRNA-IQG1-r, sgRNA-CYK3-f/sgRNA-CYK3-r, sgRNA-HOF1-f/sgRNA-HOF1-r, sgRNA-INN1-f/sgRNA-INN1-r were synthesized, annealed, and then assembled into *Bsa*I digested pZ-panARS-hCas9-sgRNA (ADE2) to give rise to plasmids pZ-panARS-hCas9-MYO1, pZ-panARS-hCas9-IQG1, pZ-panARS-hCas9-CYK3, pZ-panARS-hCas9-HOF1 and pZ-panARS-hCas9-INN1, respectively. All the plasmids were confirmed through Sanger sequencing performed by Sangon Biotech (Shanghai, China).

### Strain construction

The electroporation protocol and subsequent screening of positive transformants followed the established method Pichia Expression manuals (Invitrogen). To construct strain LYZ-A1, the plasmid pPinkHC-hLYZ was linearized using *Bcu*I and then transformed into the *Δade2*-GS115 strain. For the construction of strains LYZ-C1 and LYZ-P1, plasmids pPIC9k-PDI1 and pPIC9k were linearized with *Sac*I and transformed into strain LYZ-A1, respectively.

The gene deletion was carried out using the CRISPR/Cas9 system [31]. For gene deletion, a co-transformation was conducted using 2 µg of gRNA plasmid and 2 µg of homology arms. Positive transformants were screened on YPD plates supplemented with 100 µg/mL Zeocin (R25001, Invitrogen). The identification of positive transformants was confirmed using diagnostic PCR with the following primer sets: MYO1-TEST-f/MYO1-TEST-r, IQG1-TEST-f/IQG1-TEST-r, CYK3-TEST-f/CYK3-TEST-r, HOF1-TEST-f/HOF1-TEST-r, and INN1-TEST-f/INN1-TEST-r (Table S2). The resulting diagnostic PCR fragment was further confirmed by sanger sequencing conducted by Sangon Biotech (Shanghai, China). To amplify the homology arms of *MYO1*, *IQG1*, *CYK3*, *HOF1*, and *INN1*, two pairs of primers, MYO1-L-f/MYO1-L-r + MYO1-R-f/MYO1-R-r, IQG1-L-f/IQG1-L-r + IQG1-R-f/IQG1-R-r, CYK3-L-f/CYK31-L-r + CYK3-R-f/CYK3-R-r, HOF1-L-f/HOF1-L-r + HOF1-R-f/HOF1-R-r, INN1-L-f/INN1-L-r + INN1-R-f/INN1-R-r, were used to amplify from GS115 genomic DNA. Subsequently, the plasmids pZ-panARS-hCas9-MYO1, pZ-panARS-hCas9-IQG1, pZ-panARS-hCas9-HOF1, pZ-panARS-hCas9-CYK3, pZ-panARS-hCas9-INN1, along with their corresponding homology arms, were co-transformed into the CH2 strain, resulting in the generation of the respective gene deletion strains: *Δmyo1*-CH2, *Δiqg1*-CH2, *Δhof1*-CH2, *Δcyk3*-CH2, and *Δinn1*-CH2. To generate the strains *Δiqg1*-LYZ-C1 and *Δmyo1*-LYZ-C1, plasmids pZ-panARS-hCas9-IQG1 and pZ-panARS-hCas9-MYO1, together with their respective homology arms, were co-transformed into the strain LYZ-C1. To construct strain *Δmyo1Δiqg1*-CH2, the plasmid pZ-panARS-hCas9-MYO1 and its corresponding homology arms were co-transformed into *Δiqg1*-CH2 to generate strain *Δmyo1Δiqg1*-CH2.

### ARTP mutagenesis and screening

The strain LYZ-C1 was cultured at 30 °C to the logarithmic phase. The concentration of yeast cells was adjusted to an OD_600_ value of 0.6–0.8 using sterile water. Next, 10 µL of the yeast cell suspension was pipetted onto a steel plate and subjected to the ARTP-M mutagenesis machine (Wuxi TMAXTREE Biotechnology, Wuxi, China) at the following machine parameters: 120 W power, 10 SLM gas flow rate, and an exposure time of 120 s. Following the mutagenesis process, the steel plate was transferred to a new sterile tube containing 1 mL of sterile water, properly diluted, and grown on a YPD plate for 3–5 days. Individual colonies were selected with sterile toothpicks and dotted onto *Micrococcus lysodeikticus*-coated YPM plates. The YPM plates were then incubated at 30 °C for 48 h, and single colonies displaying large halos were chosen for further screening.

Subsequent screening was conducted using 48 deep-well plates. The seed cells were inoculated into 1 mL of BMGY medium and incubated in a shaker at 30 °C and 1000 rpm. After overnight cultivation, the deep-well plates were centrifuged, washed with sterile water twice, and resuspended in BMMY medium for induction at 30 °C and 1000 rpm. Fresh methanol was added at a final concentration of 1% (v/v) every 24 h. After 72 h of fermentation, the deep-well plates were centrifuged, and the supernatants were used for hLYZ activity assay.

### Shaking flask culture

The overnight seed cultures in BMGY were centrifuged, washed with sterile water, and subsequently resuspended in 40 mL of BMMY medium in a 250 mL shaking flask at 250 rpm and 30 °C. The initial yeast cell density in BMMY was adjusted to an OD_600_ value of 2. Fresh methanol was added to a final concentration of 1% (v/v) every 12 h. Fermentation samples were collected to measure the cell density, as well as hLYZ in both the supernatant and intracellular compartments of the yeast cells.

### Fed-batch fermentation

The recombinant strain was inoculated from a 1 mL cryostock into 200 mL of YPG medium and cultivated in a shaker at 250 rpm and 30 °C. The resulting seed cultures were then transferred to a 2 L BSM medium in a 5 L bioreactor (Baoxing, Shanghai, China), upon reaching an OD_600_ value of approximately 6–8. Following the depletion of the initial glycerol, the glycerol fed-batch was executed in accordance with the established protocols of *Pichia* fermentation (Invitrogen). After completing the glycerol fed-batch phase, methanol feeding began with the following procedure: an initial feed rate of 3 mL/h per liter fermentaion volume for 8 h, followed by an increase to 6 mL/h per liter fermentaion volume maintained throughout the remaining fermentation. The pH was regulated at 5.5 using a 25% ammonium hydroxide solution, and the temperature was maintained at 30 °C during the fermentation process. Dissolved oxygen (DO) levels were monitored and kept above 20%. Samples were collected periodically throughout the fermentation process to measure wet cell weight, hLYZ activity, and total protein concentration.

### hLYZ bioactivity assay

The activity of hLYZ was determined in accordance with the standard method specified in GB/T 30,990 − 2014. The optical density of *Micrococcus lysodeikticus* was adjusted to an OD_450_ of 1.3 using 0.1 M phosphate buffer (pH 6.2). A mixture containing 2.5 mL of *Micrococcus lysodeikticus* cell suspension and 0.5 mL of the diluted supernatant was transferred to a glass cuvette, and the change in optical density at 450 nm was measured at 25 °C. The enzyme unit was defined as the reduction in absorbance by 0.001 per minute in the reaction solution at a wavelength of 450 nm.

### Transcriptomic analysis

Yeast cells for transcriptomic analysis were cultured in BMMY medium for 36 h. Subsequently, the cells were collected and stored in liquid nitrogen. Total RNA was extracted using TRIzol Reagent according the manufacturer’s instructions. The library construction, RNA sequencing, and data analysis were carried out by Majorbio Bio-pharm Technology (Shanghai, China). The raw data is available for download from the database at https://ngdc.cncb.ac.cn/gsa [32] under the accession number CRA013498.

### Western blotting

The alkaline lysis method was utilized to prepare the total cell lysates [33]. Following this, the fermentation supernatant and total cell lysates were reduced and denatured with SDS loading buffer at 100 °C for 8 min. The protein samples were then separated by 12% SDS-PAGE and transferred onto a PVDF membrane. After being blocked with 5% skimmed milk at room temperature for 1 h, the PVDF membrane was sequentially incubated with the primary antibody and the secondary antibody (AS014, ABclonal) for 1 h each. The blots were then developed using the ECL detection kit. The primary antibodies anti-hLYZ (ab108508, Abcam) and anti-β-actin (bs8783R, Bioss) were used for the detection of hLYZ and yeast β-actin, respectively. Densitometry analysis of the blots was performed using ImageJ software.

### Fluorescence microscopy

Yeast cells for microscopy experiments were cultured in BMMY medium for 24 h. Afterward, the cells were fixed by 4% formaldehyde for 1 h at room temperature. Subsequently, the cells were washed with Phosphate-Buffered Saline (PBS) and stained with Calcofluor White (SL7204, Coolaber) at a concentration of 1 mg/mL for 10 min at room temperature. Fluorescence microscopy was carried out using a Nikon Eclipse Ci-S microscope. The contrast and brightness of the images were enhanced using NIH ImageJ.

### Flow cytometry analysis (FACS)

Yeast cells for FACS were cultured in BMMY medium for 24 h. Approximately 10^7^ yeast cells were collected through centrifuging at 2000 g for 5 minutes. The cell pellet was then fixed by resuspending it in 1 mL of 70% cold ethanol. Subsequently, the yeast cells were washed first with 1mL of 50 mM citrate buffer (pH 7.2), and then resuspended in 1 mL of citrate buffer. A final concentration of 20 µg/mL RNase A (A002131, Sangon Biotech) and 20 µg/mL propidium iodide (PI) (A601112, Sangon Biotech) was added to the cell suspension, which was incubated in the dark for 1 h. After that, the yeast cells were washed with 1 mL of citrate buffer, resuspended in an additional 1 mL of citrate buffer, and transferred to a flow tube. The yeast cell samples were briefly sonicated and analyzed using FACS (BD FACSAria II). Each sample was analyzed with over 10^5^ cells. Data analysis was performed using FlowJo™ software.

### Quantification of total protein concentration

The quantification of total protein concentration in the fermentation samples was performed using a Bradford assay kit (C503041, Sangon Biotech) following the manufacturer’s instructions. The recombinant hLYZ (L1667, Sigma) was utilized as a standard.

### *N*-glycan release and SDS-PAGE analysis

The release of *N*-glycans was achieved through digestion with the PNGase F enzyme (P0704S, NEB). The digestion protocol was followed according to the manufacturer’s instructions. The negative control group was formed by excluding the addition of the PNGase F enzyme. Subsequently, the protein samples were analyzed by SDS-PAGE and stained with Coomassie brilliant blue.

### LC-MS/MS

The 16 kDa band on the SDS-PAGE gel was excised and digested by trypsin, and then subjected to LC-MS/MS analysis using EASY-nLC 1200 (Thermo Fisher Scientific) interfaced to Q Exactive Plus mass spectrometer (Thermo Fisher Scientific). The samples were initially online-purified using an Acclaim PepMap™ 100 C18 column and then separated using an Acclaim PepMap™ RSLC C18 column. The peptide separation flow rate was set at 350 nL/min, with the following gradient: 0–3 min 1% solvent B (80% acetonitrile solution containing 0.1% formic acid), 3–73 min ramping solvent B 1–28%, 73–98 min ramping solvent B 28–45%, 98–110 min ramping solvent B 45–90%, 110–120 min maintaining solvent B at 90%. Solvent A is an aqueous solution supplemented with 0.1% formic acid.

The Orbitrap was operated with the following scan parameters: scan range, 350 to 1500 m/z; AGC target, 1 × 10^6^; resolution, 70,000. To acquire high-quality MS2 data, a data-dependent scan (dd-MS2) was employed. The top 20 most intense precursors were automatically selected for MS/MS fragmentation. The dd-MS2 parameters were as follows: resolution, 17,500; isolation window, 1.6 m/z; AGC target, 1 × 10^5^. The Normalized Collision Energy was set at 27 V.

*De novo* peptide sequencing was conducted using PEAKS X + from BioinformaticsSolutions Inc. (ON, Canada). After performing *de novo* sequencing, the results were generate by searching in the database. Glycosylation of serine/threonine (Ser/Thr) with 1 Man to 15 Man (1 M: 162.0528) was set as a variable modification. The m/z theoretical values of glycosylation modifications were then generated for every theoretical peptide backbone sequence.

### Statistical analysis

The results were presented as the means ± standard deviation of three independent experiments, unless stated otherwise. Statistical analysis was conducted using Student’s *t*-test or one-way ANOVA, as appropriate. A significance level of *p* < 0.05 was deemed to indicate a statistically significant difference.

### Electronic supplementary material

Below is the link to the electronic supplementary material.

Supplementary Material 1

## Data Availability

All data included in this study are available upon request by contact with the corresponding author.
