# Supplementary material for: Cytokinetic engineering enhances the secretory production of recombinant human lysozyme in Komagataella phaffii
Source: Microb Cell Fact. 2024 Jun 18;23:179. doi: 10.1186/s12934-024-02434-w (PMC11184742; doi:10.1186/s12934-024-02434-w)
Supplement: Supplementary file 1 — Supplementary Material 1 [file 12934_2024_2434_MOESM1_ESM.docx]

**Cytokinetic engineering enhances the secretory production of recombinant human lysozyme in *Komagataella phaffii***

Yong-Jun Zhong^#1,5^, Yang-Yang Luo ^#2,3,6^, Haiyang Xia^1,5^, Qing-Wei Zhao*^4,7^, Xu-Ming Mao*^3,4,6,7^

^1^ School of Pharmaceutical Sciences, Taizhou University, Jiaojiang 318000, Zhejiang Province, China

^2^ Polytechnic Institute, Zhejiang University, Hangzhou 310058, China

^3^ Institute of Pharmaceutical Biotechnology, School of Medicine, Zhejiang University, Hangzhou 310058, China

^4^ Department of Clinical Pharmacy, the First Affiliated Hospital & Institute of Pharmaceutical Biotechnology, School of Medicine, Zhejiang University, Hangzhou 310058, China

^5^ Zhejiang Provincial Key Laboratory of Plant Evolutionary Ecology and Conservation, Taizhou University, Taizhou 318000, China

^6^ Zhejiang Provincial Key Laboratory for Microbial Biochemistry and Metabolic Engineering, Hangzhou 310058, China

^7^ State Key Laboratory for Diagnosis and Treatment of Infectious Diseases, Hangzhou 310058, China

^#^ These two authors contributed equally to this work

Correspondence: qwzhao@zju.edu.cn (Q.-W. Z.), xmmao@zju.edu.cn (X.-M. M.)


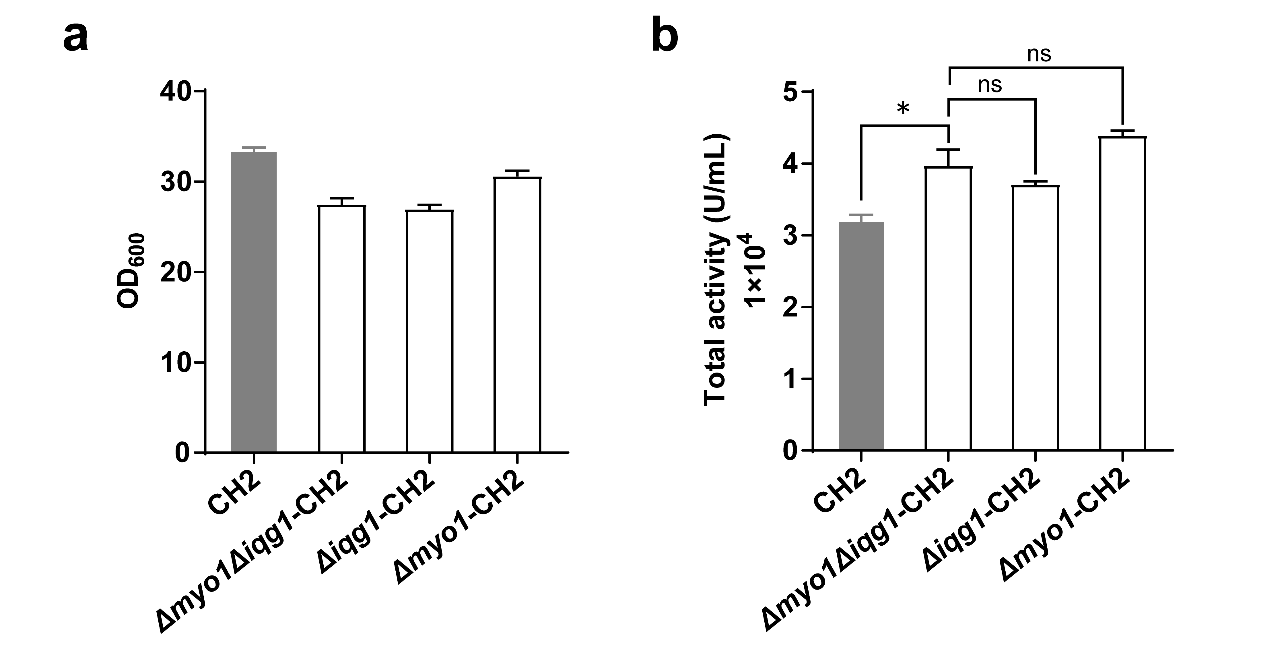


**Figure S1.** The simultaneous deletion of *MYO1* and *IQG1* genes on cell growth (a) and hLYZ bioactivity (b). ns, not significant. **p* < 0.05.


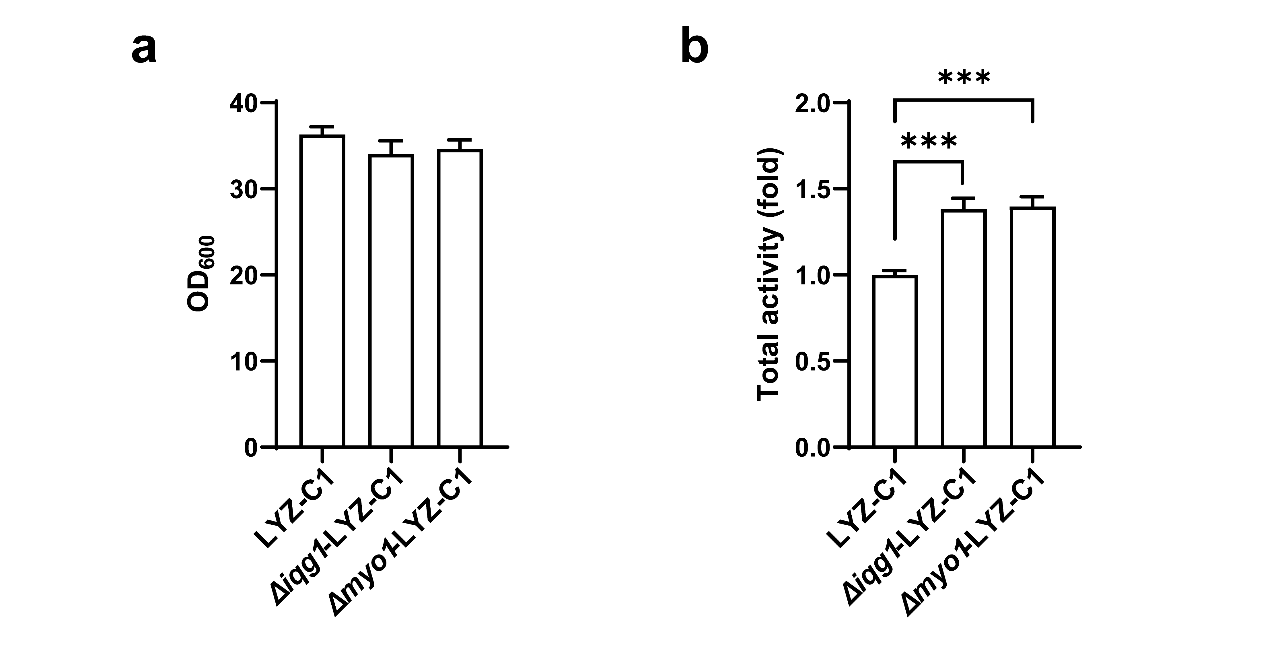


**Figure S2.** The cell growth (a) and relative hLYZ bioactivity (b) of strains LYZ-C1, *Δiqg1*- LYZ-C1 and *Δmyo1*- LYZ-C1. ****p* < 0.001.


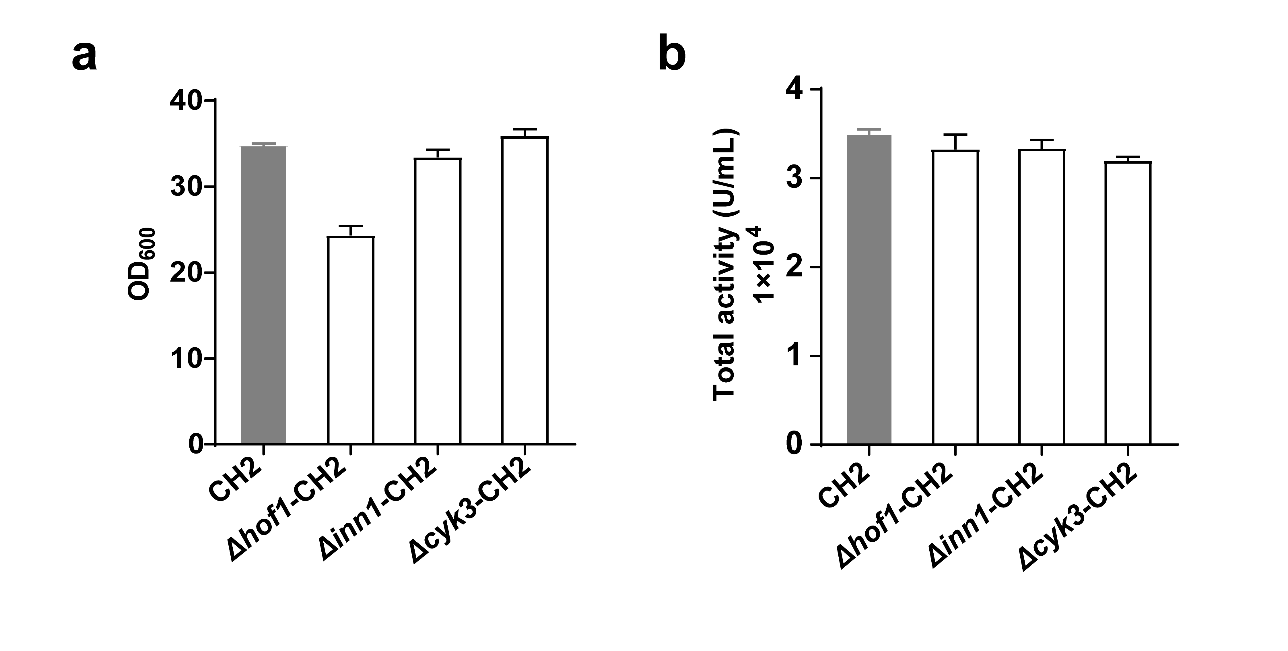


**Figure S3.** The individual deletion of *HOF1*, *INN1*, and *CYK3* genes on cell growth (a) and hLYZ bioactivity (b).





**Figure S4.** Western blotting analysis of hLYZ. The supernatant from fed-batch fermentation was examined using an anti-hLYZ antibody. Lane 1, protein marker; Lane 2, supernatant of fed-batch fermentation.





**Figure S5.** SDS-PAGE analysis of the release of *N*-linked glycosylation on hLYZ. *N*-linked glycosylation was cleaved by PNGase F and analyzed using SDS-PAGE.





**Figure S6.** Prediction of *N*-linked glycosylation sites. Potential *N*-linked glycosylation sites in hLYZ were predicted using NetNGlyc-1.0 (<https://services.healthtech.dtu.dk/services/NetNGlyc-1.0/>).





**Figure S7.** Analysis of *O*-linked glycosylation. LC-MS/MS was employed to analyze *O*-linked glycosylation in hLYZ. De novo peptide sequencing was performed in PEAKS X+. *O*-linked glycans consisting of 1, 2, 3, 4, and 6 mannose residues are represented by M1, M2, M3, M4, and M6, respectively. The decoded matching peptides are indicated by the blue line, and detailed peptide information is provided in Table S3.

**Table S1.** Strains and plasmids used in this study.

| **Strain/plasmid** | **Description** | **Sources** |
| --- | --- | --- |
| **Strains** |  |  |
| DH5α | *Escherichia coli* DH5α, | This lab |
| GS115 | Wild-type *P. pastoris strain;* Δ*his4^-^* | This lab |
| *Δade2*-GS115 | *Δade2-*GS115*;* Δ*his4^-^;* Δ*ade2^-^* | This lab |
| LYZ-A1 | GS115-Δade2 harboring plasmid pPinkHC-hLYZ | This study |
| LYZ-C1 | LYZ-A1 harboring plasmid pPIC9k-PDI1 | This study |
| LYZ-P1 | LYZ-A1 harboring plasmid pPIC9k | This study |
| CH2 | LYZ-C1 mutant of ARTP screening | This study |
| *Δiqg1*-LYZ-C1 | LYZ-C1 strain with deletion of *IQG1* gene | This study |
| *Δmyo1*- LYZ-C1 | LYZ-C1 strain with deletion of *MYO1* gene | This study |
| *Δiqg1*-CH2- | CH2 strain with deletion of *IQG1* gene | This study |
| *Δmyo1*-CH2 | CH2 strain with deletion of *MYO1* gene | This study |
| *Δcyk3*-CH2 | CH2 strain with deletion of *CYK3* gene | This study |
| *Δinn1*-CH2 | CH2 strain with deletion of *INN1* gene | This study |
| *Δhof1*-CH2 | CH2 strain with deletion of *HOF1* gene | This study |
| *Δmyo1Δiqg1*-CH2 | CH2 strain with deletion of *MYO1* and *IQG1* genes | This study |
|  |  |  |
| **Plasmids** |  |  |
| pPIC9k | *P. pastoris* expression plasmid, *HIS4* | Invitrogen |
| pPink-HC | *P. pastoris* expression plasmid, *ADE2* | Invitrogen |
| pZ-panARS-hCas9-sgRNA (ADE2) | sgRNA(*ADE2*), *hCas9*, Zeocin | a gift from prof. Jiazhang Lian |
| pPinkHC-hLYZ | Ost1-pro-hLYZ coding sequence cloned in *EcoR*I/*Kpn*I digested pPink-HC | This study |
| pPIC9k-PDI1 | *PDI1* gene cloned in *BamH*I/*Not*I digested pPIC9k | This study |
| pZ-panARS-hCas9-MYO1 | sgRNA(*MYO1*), *hCas9*, Zeocin | This study |
| pZ-panARS-hCas9-IQG1 | sgRNA(*IQG1*), *hCas9*, Zeocin | This study |
| pZ-panARS-hCas9-HOF1 | sgRNA(*HOF1*), *hCas9*, Zeocin | This study |
| pZ-panARS-hCas9-CYK3 | sgRNA(*CYK3*), *hCas9*, Zeocin | This study |
| pZ-panARS-hCas9-INN1 | sgRNA(*INN1*), *hCas9*, Zeocin | This study |

**Table S2.** Primers used for plasmid construction.

| **Name** | **Sequence (5’- 3’)** |
| --- | --- |
| hLYZ-F | ACGGAATTCAAACGATGCGCCAAGTATGGTTTAG |
| hLYZ-R | GCCGGTACCTCATTAGACTCCACAACCTT |
| PDI1-F | ACAACTAATTATTCGAAGGATCCAAACGATGCAATTCAACTGGAATAT |
| PDI1-R | AAGGCGAATTAATTCGCGGCCGCTTAAAGCTCGTCGTGAGCGT |
| sgRNA-MYO1-f | ACGCTTGCACAGAACGCAATTCAG |
| sgRNA-MYO1-r | AAACCTGAATTGCGTTCTGTGCAA |
| sgRNA-IQG1-f | ACGCTTGAGGGAATTAGAGGAACG |
| sgRNA-IQG1-r | AAACCGTTCCTCTAATTCCCTCAA |
| sgRNA-CYK3-f | ACGCTTTAGTCATATGATGTGGGA |
| sgRNA-CYK3-r | AAACTCCCACATCATATGACTAAA |
| sgRNA-HOF1-f | ACGCCAAGGGCCCAGAAACCTGAG |
| sgRNA-HOF1-r | AAACCTCAGGTTTCTGGGCCCTTG |
| sgRNA-INN1-f | ACGCTAAGTGTATTTAGTGAGCCT |
| sgRNA-INN1-r | AAACAGGCTCACTAAATACACTTA |
| MYO1-TEST-f | CACCAGCTGTCATTGGTAAGGC |
| MYO1-TEST-r | GACGATGTCTACTCACTGGCTA |
| IQG1-TEST-f | GCTTCGTCATTTATGCCAAGG |
| IQG1-TEST-r | ACCAATGCAACATGCAAACC |
| CYK3-TEST-f | CCTAAGAGCTTCCCTACCAGA |
| CYK3-TEST-r | CTCGCATTACCTTAGAGTGTGT |
| HOF1-TEST-f | GAGAGCATTGAGGATGAGGTCA |
| HOF1-TEST-r | ACGGTTACTGGTAGCTTCAAGG |
| INN1-TEST-f | GGGTGATGGGGAACACTGAAA |
| INN1-TEST-r | ATTGTATGATGTAATGGGAGTTGAC |
| MYO1-L-f | GTTAATTAGGACTCTTGCTTTTTGA |
| MYO1-L-r | TTAAATATACCATGTACATATCTTTGTACAAGTCGGCAATG |
| MYO1-R-f | CATTGCCGACTTGTACAAAGATATGTACATGGTATATTTAACGACG |
| MYO1-R-r | TAAAGTTGTTGCAGAACTCTTAGG |
| IQG1-L-f | GTGTACAATGTCACTCTTAATC |
| IQG1-L-r | TAGTTTCTTGGTACATATTTTGAACAATGTGAAAGTGATGTG |
| IQG1-R-f | CACATCACTTTCACATTGTTCAAAATATGTACCAAGAAACTA |
| IQG1-R-r | CTTCCACTGGTGGTAACTGT |
| CYK3-L-f | CTGCTGTTGTCATGTAATACGA |
| CYK3-L-r | ACCTTTACTCCTCTCTATTGATAAAGCATTATATAGTGCATATAATAAAC |
| CYK3-R-f | TGCACTATATAATGCTTTATCAATAGAGAGGAGTAAAGGTTGG |
| CYK3-R-r | GGGAAGTGAGAAGTCACAAAG |
| HOF1-L-f | GGAAGCGCAAGGGTGATATG |
| HOF1-L-r | TCAGTTCTTGAATCCCCAAGGGTATATATTACATTACAAAAGGTGGT |
| HOF1-R-f | TTTGTAATGTAATATATACCCTTGGGGATTCAAGAACTGAG |
| HOF1-R-r | GCTGGCGGCAAGTTTGGCTA |
| INN1-L-f | CCCAACACGAATTGACAGAA |
| INN1-L-r | TTATTATTAGTACGTTCTATTTGTTTTGAAAATCACGAGAAATAG |
| INN1-R-f | TCTCGTGATTTTCAAAACAAATAGAACGTACTAATAATAATATTAAAGAC |
| INN1-R-r | CCTCTTCCTATCAATGGTCCC |

**Table S3.** Supporting peptides of LC-MS/MS

| **Peptide** | **Mass** | **ppm** | **m/z** | **RT**  **/min** | **PTM** |
| --- | --- | --- | --- | --- | --- |
| ATNYNAGDRSTDYGIFQINSR | 2362.094 | -1.3 | 1182.053 | 58.13 |  |
| TNYNAGDRSTDYGIFQINSR | 2291.057 | -2.6 | 764.6909 | 58.05 |  |
| STDYGIFQINSR | 1399.673 | 0.4 | 700.8442 | 64.02 |  |
| SALLQDNIADAVAC(+57.02)AK | 1658.83 | 2.2 | 830.424 | 66.94 | Carbamidomethylation |
| DRSTDYGIFQINSR | 1670.801 | -1.7 | 836.4065 | 59.43 |  |
| TPGAVNAC(+57.02)HLSC(+57.02)SALLQDNIADAVAC(+57.02)AKR | 3082.475 | 0.4 | 771.6262 | 65.34 | Carbamidomethylation |
| VVRDPQGIRAWVAWR | 1807.996 | 0.6 | 453.0065 | 56.03 |  |
| GISLANWMC(+57.02)LAK | 1362.679 | 0.3 | 682.3469 | 80.33 | Carbamidomethylation |
| LGM(+15.99)DGYRGISLANWMC(+57.02)LAK | 2171.033 | 1.3 | 724.6857 | 78.42 | Oxidation (M); Carbamidomethylation |
| WESGYNTRATNYNAGDR | 1973.862 | 0.4 | 658.9614 | 38.96 |  |
| YNAGDRSTDYGIFQINSR | 2075.966 | -1 | 692.9952 | 58.36 |  |
| TDYGIFQINSR | 1312.641 | -0.9 | 657.3273 | 65.53 |  |
| LGM(+15.99)DGYRGISLANWM(+15.99)C(+57.02)LAK | 2187.028 | -0.4 | 730.0162 | 70.66 | Oxidation (M); Carbamidomethylation |
| VFERC(+57.02)ELARTLKR | 1676.914 | 0.2 | 420.236 | 35.89 | Carbamidomethylation |
| VVRDPQGIRAW(+15.99)VAWR | 1823.991 | 1.3 | 457.0056 | 45.31 | Oxidation (HW) |
| NYNAGDRSTDYGIFQINSR | 2190.009 | -0.5 | 731.0099 | 58.36 |  |
| TPGAVNAC(+57.02)HLSC(+57.02)SALLQDNIADAVAC(+57.02)AK | 2926.374 | -0.2 | 976.465 | 69.91 | Carbamidomethylation |
| C(+57.02)QNRDVRQYVQGC(+57.02)GV | 1837.831 | -0.9 | 919.922 | 43.18 | Carbamidomethylation |
| VFERC(+57.02)ELAR | 1178.587 | 2.1 | 393.8703 | 33.21 | Carbamidomethylation |
| RVVRDPQGIR | 1194.695 | 1.2 | 399.2393 | 25.69 |  |
| GISLANWM(+15.99)C(+57.02)LAK | 1378.674 | 0.4 | 690.3444 | 70.02 | Oxidation (M); Carbamidomethylation |
| NAGDRSTDYGIFQINSR | 1912.903 | -0.6 | 638.6411 | 56.29 |  |
| GAVNACHLSC(+57.02)SALLQDNIADAVAC(+57.02)AK | 2671.252 | 1.9 | 891.4262 | 68.44 | Carbamidomethylation |
| GISLANW(+15.99)MC(+57.02)LAK | 1378.674 | 0.4 | 690.3444 | 70.02 | Oxidation (HW); Carbamidomethylation |
| KVFERC(+57.02)ELARTLKR | 1805.009 | 1.6 | 452.2603 | 34.1 | Carbamidomethylation |
| TLKRLGMDGYR | 1308.697 | 2.7 | 437.2409 | 33 |  |
| ATNYNAGDRSTDYGIF | 1763.775 | -1.1 | 882.8938 | 61.03 |  |
| ATNYNAGDR | 980.4312 | 0 | 491.2228 | 25.15 |  |
| GDRSTDYGIFQINSR | 1727.823 | -0.1 | 576.9481 | 55.89 |  |
| C(+57.02)HLSC(+57.02)SALLQDNIADAVAC(+57.02)AK | 2316.066 | -1.8 | 773.028 | 66.13 | Carbamidomethylation |
| KVFERC(+57.02)ELAR | 1306.682 | -0.4 | 654.3478 | 28.58 | Carbamidomethylation |
| ATNYNAGDRSTDYG | 1503.623 | 1.4 | 752.8196 | 34.08 |  |
| RLGMDGYR | 966.4705 | 0.1 | 484.2426 | 32.04 |  |
| NIADAVAC(+57.02)AKR | 1187.608 | -0.6 | 396.8764 | 35.54 | Carbamidomethylation |
| YGIFQINSR | 1096.567 | 0.8 | 549.291 | 57.83 |  |
| DVRQYVQGC(+57.02)GV | 1279.598 | 0 | 640.8062 | 54.61 | Carbamidomethylation |
| ATNYNAGDRSTDY | 1446.601 | -0.7 | 724.3073 | 34.61 |  |
| AC(+57.02)HLSC(+57.02)SALLQDNIADAVAC(+57.02)AKR | 2543.204 | -0.1 | 636.8083 | 61.07 | Carbamidomethylation |
| LGMDGYR | 810.3694 | 0.3 | 406.1921 | 39.65 |  |
| ATNYNAGDRSTD | 1283.538 | -1.1 | 642.7755 | 26.96 |  |
| C(+57.02)SALLQDNIADAVAC(+57.02)AKR | 1974.962 | 2.6 | 659.3295 | 60.48 | Carbamidomethylation |
| KVFERCELAR | 1249.66 | -1.5 | 417.56 | 34.63 |  |
| VVRDPQGIR | 1038.593 | -1.2 | 520.3033 | 26.92 |  |
| SC(+57.02)SALLQDNIADAVAC(+57.02)AKR | 2061.993 | -0.6 | 688.338 | 61.73 | Carbamidomethylation |
| SALLQDNIADAVAC(+57.02)AKR | 1814.931 | -1 | 605.9836 | 59.53 | Carbamidomethylation |
| AGDRSTDYGIFQINSR | 1798.86 | -1.2 | 600.6265 | 55.69 |  |
| SC(+57.02)SALLQDNIADAVAC(+57.02)AK | 1905.893 | 1 | 953.9545 | 68.73 | Carbamidomethylation |
| DYGIFQINSR | 1211.594 | 1 | 606.8046 | 67.88 |  |
| LGM(+15.99)DGYR | 826.3643 | 1.6 | 414.1901 | 29.85 | Oxidation (M) |
| ATNYNAGDRST(+162.05)D | 1445.591 | 18.6 | 723.816 | 31.82 | M1 |
| NRC(+57.02)QNRDVRQYVQGC(+57.02)GV | 2107.975 | -0.4 | 703.6653 | 37.75 | Carbamidomethylation |
| YWC(+57.02)NDGK | 941.3701 | 1.4 | 471.693 | 30.3 | Carbamidomethylation |
| WESGYNTR | 1011.441 | 1.2 | 506.7284 | 28.46 |  |
| ATNYNAGDRSTDYGIFQIN | 2118.961 | 1.5 | 1060.489 | 68.06 |  |
| LQDNIADAVAC(+57.02)AK | 1387.677 | 1.4 | 694.8465 | 51.91 | Carbamidomethylation |
| DNIADAVAC(+57.02)AK | 1146.534 | -0.1 | 574.2742 | 47.9 | Carbamidomethylation |
| GIFQINSR | 933.5032 | -0.2 | 467.7588 | 48.5 |  |
| FERC(+57.02)ELAR | 1079.518 | 22.8 | 360.8549 | 33.41 | Carbamidomethylation |
| TPGAVNAC(+57.02)HLS | 1125.524 | -0.1 | 563.769 | 33.92 | Carbamidomethylation |
| LGMDGYRGISLAN | 1365.671 | 0 | 683.8428 | 63.59 |  |
| RDVRQYVQGC(+57.02)GV | 1435.699 | 0.7 | 479.5739 | 42.33 | Carbamidomethylation |
| RVVRDPQGIRAWVAWR | 1964.097 | 1.7 | 492.0323 | 51.08 |  |
| C(+57.02)ELARTLKR | 1145.634 | -3.1 | 382.8841 | 27.09 | Carbamidomethylation |
| LSC(+57.02)SALLQDNIADAVAC(+57.02)AKR | 2175.078 | 1.7 | 726.0344 | 67.82 | Carbamidomethylation |
| RDPQGIR | 840.4565 | -2.2 | 421.2346 | 26.92 |  |
| VRDPQGIR | 939.525 | -0.9 | 470.7693 | 26.92 |  |
| IFQINSR | 876.4818 | -0.8 | 439.2478 | 40.51 |  |
| RATNYNAGDRSTDYGIFQINSR | 2518.195 | 0.7 | 630.5564 | 50.82 |  |
| QDNIADAVAC(+57.02)AK | 1274.593 | -0.6 | 638.3032 | 45.5 | Carbamidomethylation |
| NIADAVAC(+57.02)AK | 1031.507 | 0 | 516.7607 | 40.32 | Carbamidomethylation |
| TRATNYNAGDRSTDYGIFQINSR | 2619.242 | 0.3 | 655.8181 | 51.08 |  |
| LGMDGYRG | 867.3909 | -1.4 | 434.7021 | 39.07 |  |
| IADAVAC(+57.02)AKR | 1073.565 | 1.4 | 358.8629 | 27.26 | Carbamidomethylation |
| S(+974.32)TDYGIFQINSR | 2373.99 | 43.6 | 792.3718 | 59.01 | M6 |
| DNIADAVAC(+57.02)AKR | 1302.635 | 0.6 | 652.3252 | 41.85 | Carbamidomethylation |
| C(+57.02)ELARTLK | 989.5328 | -1.1 | 495.7731 | 28.5 | Carbamidomethylation |
| ERC(+57.02)ELAR | 932.4498 | -0.2 | 467.2321 | 33.21 | Carbamidomethylation |
| VFERC(+57.02)ELA | 1022.486 | 1.3 | 512.2507 | 40.68 | Carbamidomethylation |
| LGM(+15.99)DGYRGISLAN | 1381.666 | -0.1 | 691.8402 | 53.59 | Oxidation (M) |
| RS(+324.11)T(+486.16)DYGIFQINSR | 2366.038 | 18 | 592.5275 | 55.18 | M2; M3 |
| KW(+15.99)ESGYNTR | 1155.531 | -19.7 | 578.7614 | 30.44 | Oxidation (HW) |
| GDRS(+162.05)T(+486.16)DYGIFQINSR | 2376.034 | 12.8 | 793.0287 | 58.16 | M1; M3 |
| NACHLSCSALLQDNIADAVAC(+57.02)AK | 2387.103 | 2.7 | 796.7105 | 69.08 | Carbamidomethylation |
| RQYVQGC(+57.02)GV | 1065.503 | 2.8 | 533.7601 | 32.51 | Carbamidomethylation |
| LSC(+57.02)SALLQDNIADAVAC(+57.02)AK | 2018.976 | 1.7 | 1010.497 | 74.54 | Carbamidomethylation |
| DVRQYVQGC(+57.02) | 1123.508 | 0.1 | 562.7614 | 47.01 | Carbamidomethylation |
| HLSC(+57.02)SALLQDNIADAVAC(+57.02)AK | 2156.035 | 0.4 | 719.686 | 65.55 | Carbamidomethylation |
| NRC(+57.02)QNRDVRQYVQGCG | 1951.885 | -1.2 | 651.6349 | 32.24 | Carbamidomethylation |
| YNAGDRST(+324.11)DYGIFQINSR | 2400.072 | -15.9 | 601.0157 | 58.13 | M2 |
| KTPGAVNAC(+57.02)HLSC(+57.02)SALLQDNIADAVAC(+57.02)AK | 3054.469 | -0.3 | 764.6242 | 63.25 | Carbamidomethylation |
| ADAVACAKR | 903.4596 | 19.7 | 452.746 | 81.17 |  |
| STDYGIF | 801.3545 | 2.9 | 802.3641 | 71.61 |  |
| C(+57.02)QNRDVRQY | 1237.562 | 0.8 | 413.5283 | 25.53 | Carbamidomethylation |
| KVFERC(+57.02)ELARTLK | 1648.908 | 0.9 | 550.6439 | 34.27 | Carbamidomethylation |
| RC(+57.02)QNRDVRQYVQGC(+57.02)GV | 1993.932 | 1.3 | 499.491 | 37.59 | Carbamidomethylation |
| W(+15.99)ESGYNTR | 1027.436 | 0.7 | 514.7256 | 30.88 | Oxidation (HW) |
| DVRQYVQ | 906.4559 | 1.9 | 454.2361 | 40.89 |  |
| HLSC(+57.02)SALLQDNIADAVAC(+57.02)AKR | 2312.137 | 0.2 | 579.0415 | 60.26 | Carbamidomethylation |
| NAGDRST(+486.16)DYGIFQINSR | 2399.061 | -11 | 480.8142 | 58.1 | M3 |
| QYVQGC(+57.02)GV | 909.4014 | -0.1 | 455.7079 | 40.56 | Carbamidomethylation |
| GISLANW(+15.99)M(+15.99)C(+57.02)LAK | 1394.669 | -0.5 | 698.3412 | 64.86 | Oxidation (HW); Oxidation (M); Carbamidomethylation |
| DVRQYVQG | 963.4774 | -187.5 | 482.6556 | 43.53 |  |
| IADAVAC(+57.02)AK | 917.4641 | 1.2 | 459.7399 | 29.43 | Carbamidomethylation |
| C(+57.02)QNRDVRQYVQGCG | 1681.741 | 1.4 | 561.5885 | 36.84 | Carbamidomethylation |
| VVRDPQGIRA | 1109.631 | 0.6 | 555.8229 | 28.58 |  |
| LGMDGYRGISLA | 1251.628 | -0.4 | 626.8211 | 65.53 |  |
| VVRDPQGIRAWVAWRNR | 2078.14 | 1.3 | 416.6358 | 49.51 |  |
| VFERC(+57.02)ELARTLK | 1520.813 | 1.1 | 507.9456 | 38.24 | Carbamidomethylation |
| STDYGIFQIN | 1156.54 | 1.2 | 1157.549 | 81.36 |  |
| ATNYNAGDRSTDYGIFQ | 1891.834 | 0.9 | 946.9249 | 58.73 |  |
| LLQDNIADAVAC(+57.02)AK | 1500.761 | 1 | 751.3883 | 62.29 | Carbamidomethylation |
| ATNYNAGDRSTDYGIFQINSRY | 2525.157 | 2.4 | 842.7283 | 63.59 |  |
| LGM(+15.99)DGYRGISLANW(+15.99)MC(+57.02)LAK | 2187.028 | 0.8 | 730.0171 | 69.33 | Oxidation (M); Oxidation (HW); Carbamidomethylation |
| ATNYNAGDRS(+486.16)TDYGIFQ | 2377.992 | 39.2 | 793.7024 | 54.97 | M3 |
| ATNYNAGDRST(+486.16)DYGIFQ | 2377.992 | 39.2 | 793.7024 | 54.97 | M3 |
| VFERC(+57.02)EL | 951.4484 | 1 | 476.7319 | 41.56 | Carbamidomethylation |
| YWC(+57.02)NDGKTPGAVNA | 1551.678 | 0.1 | 776.8462 | 44.77 | Carbamidomethylation |
| AC(+57.02)HLSC(+57.02)SALLQDNIADAVAC(+57.02)AK | 2387.103 | -0.1 | 796.7083 | 66.29 | Carbamidomethylation |
| NRCQNRDVRQYVQGC(+57.02)GV | 2050.954 | 36.2 | 513.7643 | 45.28 | Carbamidomethylation |
| VRQYVQGC(+57.02)GV | 1164.571 | 1.6 | 583.2936 | 35.26 | Carbamidomethylation |
| RYW(+15.99)CNDGKTPGAVNACHLSCSALLQDNIADAVAC(+57.02)AKR | 4006.866 | -5.1 | 802.3763 | 66.68 | Oxidation (HW); Carbamidomethylation |
| YVQGC(+57.02)GV | 781.3429 | -0.7 | 391.6785 | 35.54 | Carbamidomethylation |
| AKWESGYNTR | 1210.573 | 0.5 | 606.2941 | 29.7 |  |
| DVRQYVQGCG | 1123.508 | 0.1 | 562.7614 | 47.01 |  |
| LGMDGYRGI | 980.4749 | -0.2 | 491.2447 | 53.59 |  |
| ATNYNAGDRS(+648.21)TDYGIFQINSRYWCNDGK | 3876.643 | 1.6 | 1293.224 | 66.96 | M4 |
| KTPGAVNACHLSCSALLQDNIADAVAC(+57.02)AKR | 3096.527 | -10.1 | 775.1311 | 65.76 | Carbamidomethylation |
| RVVRDPQGIRA | 1265.732 | 0.8 | 422.9182 | 27.15 |  |
| LGMDGYRGISLANW(+15.99)M(+15.99)C(+57.02)LAK | 2187.028 | 2.7 | 730.0184 | 66.28 | Oxidation (HW); Oxidation (M); Carbamidomethylation |
